# Vegetarian diets, circulating miRNA expression and healthspan in subjects living in the Blue Zone

**DOI:** 10.1093/pcmedi/pbaa037

**Published:** 2020-10-23

**Authors:** Tiantian Liu, Nicole M Gatto, Zhong Chen, Hongyu Qiu, Grace Lee, Penelope Duerksen-Hughes, Gary Fraser, Charles Wang

**Affiliations:** Center for Genomics, School of Medicine, Loma Linda University, Loma Linda, CA 92350, USA; School of Community and Global Health, Claremont Graduate University, Claremont, CA 91711, USA; Center for Genomics, School of Medicine, Loma Linda University, Loma Linda, CA 92350, USA; Center of Molecular and Translational Medicine, Institution of Biomedical Science, Georgia State University, Atlanta, GA 30303, USA; Department of Psychology, School of Behavioral Health, Loma Linda University, Loma Linda, CA 92350, USA; Department of Basic Sciences, School of Medicine, Loma Linda University, Loma Linda, CA 92350, USA; School of Public Health, Loma Linda University, Loma Linda, CA 92350, USA; Center for Genomics, School of Medicine, Loma Linda University, Loma Linda, CA 92350, USA; Department of Basic Sciences, School of Medicine, Loma Linda University, Loma Linda, CA 92350, USA

**Keywords:** circulating miRNA, aging, miRNA sequencing, vegetarian diet, Blue Zone

## Abstract

A long-term vegetarian diet plays a role in the longevity and maintenance of the healthspan, but the underlying mechanisms for these observations are largely unknown. Particularly, it is not known whether a long-term vegetarian dietary pattern may affect the circulating miRNA expression in such a way as to modulate the healthspan. The Adventist Health Study-2 (AHS-2) cohort includes a large number of older adults who primarily follow vegetarian dietary patterns and reside in Loma Linda, California, one of five “Blue Zones” in the world in which a higher proportion of the population enjoys a longer than average lifespan. We performed miRNA-seq in 96 subjects selected from the AHS-2 cohort with different dietary patterns. We identified several differentially expressed miRNAs between vegetarians and non-vegetarians, which are involved in immune response and cytokine signaling, cell growth and proliferation as well as age-related diseases such as cardiovascular diseases and neurodegenerative diseases. Overall, our study showed that a vegetarian diet modulates aging-associated circulating miRNAs in a sex-dependent manner of differential expression for certain miRNAs, which may be related in a beneficial manner to the healthspan. Further investigation is needed to validate these miRNAs as potential biomarkers for diet-modulated longevity in humans.

## Introduction

Aging is a complicated process that may be regulated by a combination of genetic factors, external environmental factors, and lifestyle factors such as diet and exercise, which modify epigenetic patterns including DNA methylation and chromatin remodeling.^1^ With dietary and caloric restriction shown to be effective interventions to increase lifespan in animal models and humans, interest in the relationships between diet and aging has grown.^2,3^ Although the molecular mechanisms underlying how diet affects lifespan and healthspan (commonly defined by the period of life spent in good health, free from chronic diseases and disabilities associated with aging) are not thoroughly understood, nutrient-sensing pathways are thought to play an important role.^4,5^ Nutrient-sensing pathways are used by organisms to respond to, and utilize, organic molecules such as glucose, lipids, and amino acids, in order to generate energy or cellular components.^6,7^ Pathways such as the insulin/insulin-like growth factor (IGF-1) pathway, the mechanistic target of the rapamycin (mTOR) pathway, AMP kinase signaling, and sirtuin signaling pathways have been studied in animals and humans for their links between diet and aging processes.^8–12^ Through coordination, this network of pathways is able to regulate cell inflammation, autophagy, senescence, and metabolism in response to nutrients, which in turn affects aging.^4,13^

With a large Seventh-Day Adventist population, Loma Linda, California, is one of the five “Blue Zones” in the world where people experience a longer than average life expectancy.^14^ A high proportion of Adventist Health Study-2 (AHS-2) participants follow vegetarian dietary patterns, which may provide valuable insight into the impact of diet on longevity/healthspan.^15,16^ The AHS-2 is a large (N = 96 000), well-characterized cohort with extensive follow-up that began in 2002, led by researchers at Loma Linda University (LLU).^17^ The AHS studies have advanced our understanding of the impact of diet on longevity, health, and disease. Previous studies, including those among the AHS-2, suggest that vegetarian dietary patterns may be associated with an increased lifespan and a decreased risk of age-related diseases, but results have been mixed.^16,18–21^ Although there are numerous dietary and lifestyle intervention studies in aging populations, because of the lack of an integrative marker to quantify the impact of diet, most of these studies have been observational and provide little biological information.^22,23^ The focus on epigenetic regulators of aging has attracted increasing attention to the regulation of microRNAs (miRNAs).^24^ Because these molecules target multiple age-related biological processes or pathways, for example nutrient-sensing pathways, miRNA regulation is considered to be an important aspect of the aging process in organisms.^25^ Changes in miRNA expression profiles with age occur in multiple organisms including humans, implying that miRNA, and circulating miRNAs in particular, have great potential as biomarkers for aging and aging-associated diseases.^26^ Additionally, previous studies document sexual dimorphisms in gene expression as well as in miRNA expression, which contribute to sex differences in multiple physiological and pathological processes.^27,28^ If not factored in, sex-based differences may influence the profiling of diet-induced miRNA regulation and compromise the accuracy of potential circulating biomarkers.

In searching for potential biomarkers to monitor the effect of vegetarian diets on human healthspan and longevity and to investigate healthspan and lifestyle associations at a molecular level, we aimed to identify a range of circulating miRNAs that are differentially expressed among AHS-2 participants with different dietary patterns using deep miRNA sequencing. Additionally, by comparing miRNA profiles between men and women, we sought to examine the influence of sexual dimorphisms on diet-induced circulating miRNA regulation. To take into consideration that study participants may have switched dietary patterns in the recent decade resulting in differential stability of diet among subjects, we also evaluated the influence of adherence to dietary patterns on circulating miRNA expression. Our study showed that a vegetarian diet modulates aging-associated circulating miRNAs in a sex-dependent manner of differential expression for certain miRNAs, which may be related in a beneficial manner to healthspan.

## Results

### Study participants’ characteristics

Characteristics of the study population are summarized in Table 1. Based on reported dietary habit at the time of blood draw, there were 31 non-vegetarians (NV), 15 vegans, 32 lacto-vegetarians (lacto), and 18 semi-vegetarians (semi) [collectively vegetarians (V)]. There were no differences in sex, age, and BMI of participants by dietary group (*P* > 0.05). Adherence was an independent variable (*P* = 0.03), where vegetarians had a higher proportion of switchers than non-vegetarians.

**Table 1.** Subject characteristics by current dietary pattern.

Characteristics	NV	V				*P* ^a^
		Lacto	Semi	Vegan	*P* ^b^	
Sample size (N)	31	32	18	15		
Age at baseline (years, M ± SD)	61.6 ± 12.8	58.3 ± 9.2	65.6 ± 10.4	59.8 ± 10.4	0.27	0.72
BMI (kg/m^2^, M ± SD)	26.0 ± 3.7	25.9 ± 4.1	25.5 ± 6.1	28.9 ± 4.9	0.06	0.28
Male (N, %)	16 (51.6%)	14 (43.8%)	7 (38.9%)	6 (40.0%)	0.80	0.35
Adherer (N, %)	23 (74.2%)	23 (71.9%)	7 (38.9%)	3 (20%)	0.0006	0.03

NV: Non-vegetarians, V: Vegetarians, Lacto: Lacto-ovo-vegetarians, Semi: Semi-vegetarians. BMI: body-mass index.

a ANOVA (for age and BMI) or Chi-square tests (for male and adherer) between V and NV.

b ANOVA (for age and BMI) or Chi-square tests (for male and adherer) among lacto, semi, vegan and NV. The null hypothesis for all significance tests is that there was no difference in stated parameters between dietary groups.

### miRNA-seq and mapping QC

**Figure 1.**
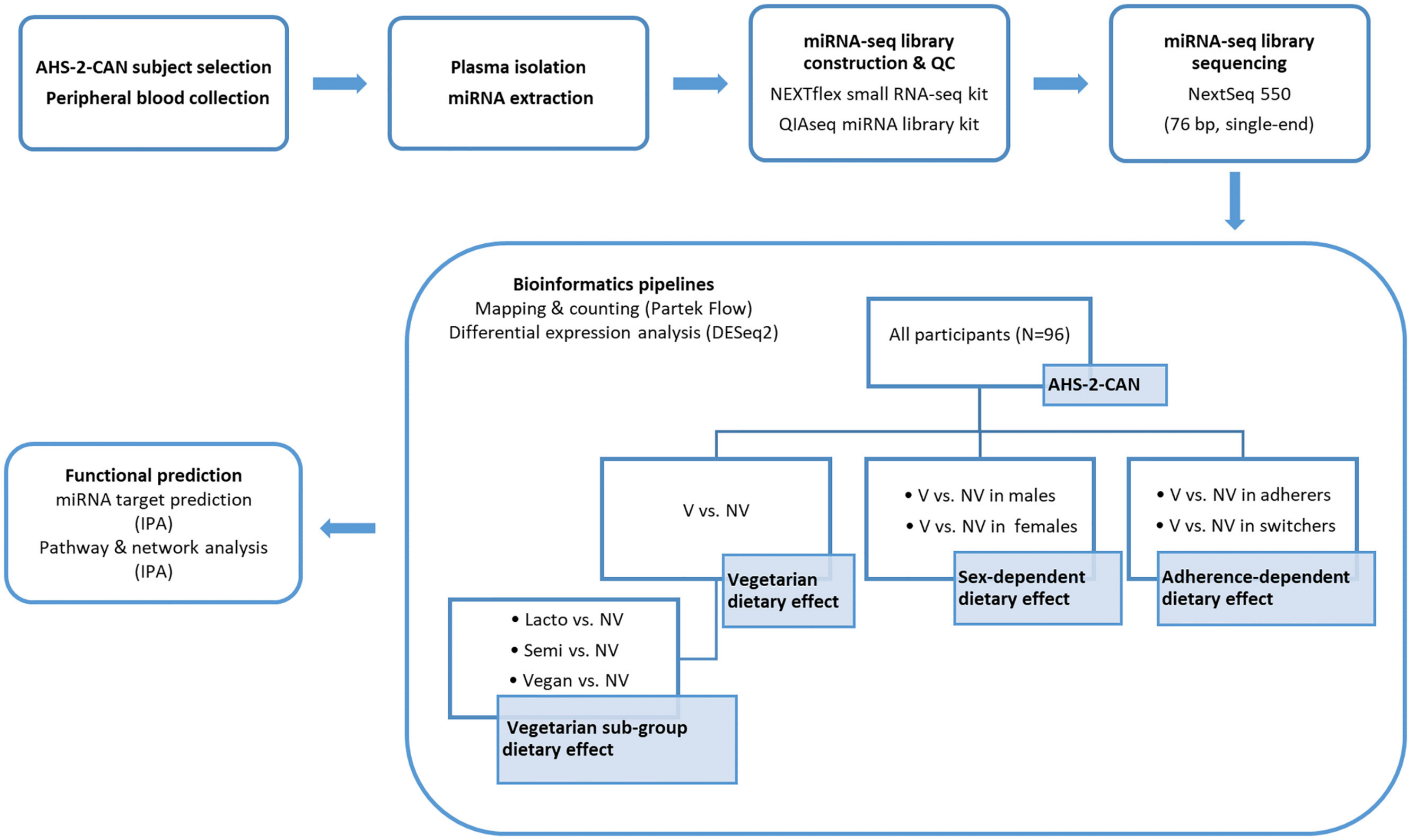
Overall study design. Peripheral blood samples from 96 AHS-2-CAN (AHS-2 Cognitive and Neuroimaging) participants included in the current study were collected. Following plasma isolation, miRNAs were extracted and miRNA-seq libraries were constructed. QC analysis and quantification were performed on all miRNA-seq libraries before sequencing. After sequencing, the reads were aligned and counted using Partek Flow miRNA-seq pipeline. Multiple differential analyses were performed to investigate the influences of specific dietary pattern, sex, and adherence to diet, respectively, on dietary regulation on the circulating miRNA expression. Functional prediction including miRNA target prediction and pathway/network enrichment analyses were conducted using the differentially expressed miRNAs identified.

**Figure 2.**
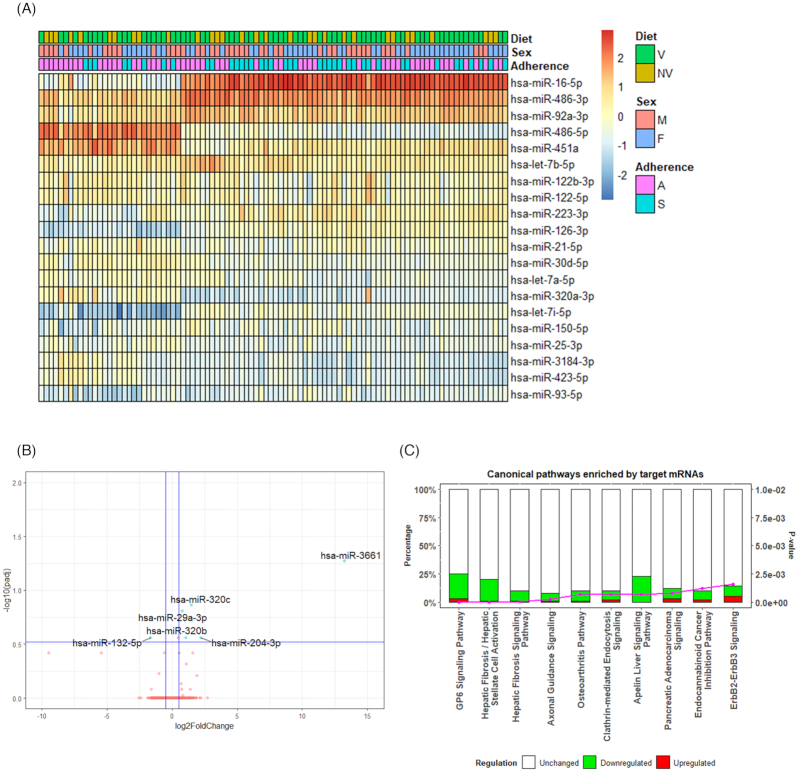
Most abundant miRNAs across all samples and differentially expressed miRNAs between vegetarians and non-vegetarians. (**A**) Heat map showing the top 20 most abundant miRNAs across all samples; (**B**) Volcano plot showing the differentially expressed miRNAs between V and NV groups. The Y-axis represents the -log10(padj) and the X-axis displays the log2FC value. A positive x-value represents an upregulation and a negative x-value represents a downregulation compared to NV. –log10(padj) ≥ 0.52 (padj ≤ 0.3) and |log2FC| ≥ 0.5 were marked as the significant threshold. Each dot represents one differentially expressed miRNA, with those above the significance threshold highlighted in blue. Significant differentially expressed miRNAs are annotated in the plot. V: Vegetarians; NV: Non-vegetarians. (**C**) Bar graphs showing the top 10 enriched canonical pathways by IPA (V vs. NV). Y-axis^1^ (left) represents the percentage of regulated genes to all genes in the pathway. Y-axis^2^ (right) represents the *P*-value of the enrichment. Downregulated genes are colored in green, upregulated genes are colored in red and unchanged genes are colored in white. V: vegetarians; NV: non-vegetarians, M: males; F: females; A: adherers; S: switchers.

miRNA-seq was carried out on all 96 samples (Fig. 1). The average number of input reads was 17 million per sample. After trimming and filtering, approximately 45% of the reads were aligned to the hg38 by Bowtie 2 aligner with average 5X coverage for each nucleotide position. Approximately 15% of all reads were mapped to miRBase version 22. The QC data are listed in Supplementary Figure 1. A total of 1364 miRNAs was detected across all 96 samples. The top 20 most abundant miRNAs detected are shown in Figure 2A.

### Differential circulating miRNA expression between vegetarians and non-vegetarians

Six significant differentially expressed (DE) miRNAs (DE miRNAs) were identified between V and NV groups (padj ≤ 0.3, |log2FC| ≥ 0.5) with five upregulated and one downregulated (Supplementary Table 1). Figure 2B displays a volcano plot showing the log2 fold change (log2FC) of each miRNA plotted against its respective FDR adjusted *P*-value (padj). As highlighted in the volcano plot, significant DE miRNAs were miR-3661 (padj = 0.05, log2FC = 13.26), miR-320c (padj = 0.14, log2FC = 1.48), miR-29a-3p (padj = 0.16, log2FC = 0.78), miR-320b (padj = 0.27, log2FC = 1.08), and miR-132-5p (padj = 0.27, log2FC = −1.64), ranging from −1.64 to 13.6 in log2FC. The ingenuity pathway analysis (IPA) miRNA target analysis identified 937 mRNAs targeted by the significant DE miRNAs in the V compared with NV group (Supplementary Table 2). The IPA core analysis of these mRNAs identified 96 significant enriched canonic pathways using the experimentally observed IPA database (Supplementary Table 3). The 10 most significant pathways are shown in Figure 2C, which included the GP6 signaling, hepatic fibrosis signaling, axonal guidance signaling and osteoarthritis pathways.

### Differential circulating miRNA expression in different sub-types of vegetarians compared to non-vegetarians

**Figure 3.**
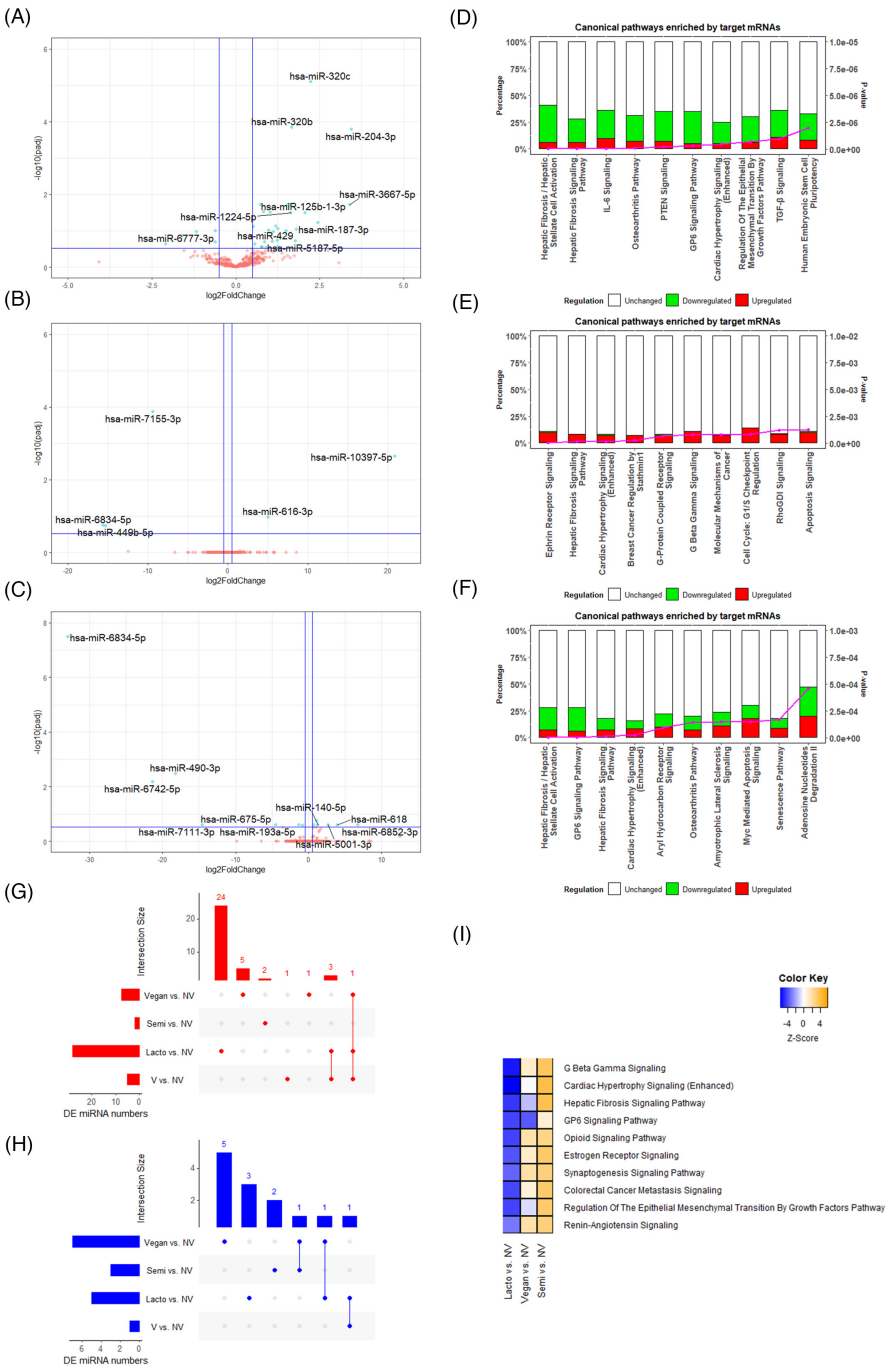
Differentially expressed miRNAs in lacto, semi, and vegan vegetarians compared to NV. Volcano plots showing the differentially expressed miRNAs between (**A**) Lacto and NV; (**B**) Semi and NV; and (**C**) Vegan and NV. –log10(padj) ≥ 0.52 (padj ≤ 0.3) and |log2FC| ≥ 0.5 were marked as the significant threshold. The significant DE miRNAs (top 10 DE miRNA by |log2FC| if more than 10 significant DE miRNAs present) are highlighted. Bar graphs showing the top 10 enriched canonical pathways between (**D**) Lacto and NV; (**E**) Semi and NV; (**F**) Vegan and NV. UpSet plots showing the number of significantly (**G**) upregulated; and (**H**) downregulated DE miRNAs across four comparisons (Lacto vs. NV, Semi vs. NV, Vegan vs. NV and V vs. NV in general), and the overlapping between each set of miRNAs. (**I**) Most distinctive enrichments in canonical pathway among three vegetarian subgroups. V: vegetarians, NV: non-vegetarians, Lacto: lacto-ovo-vegetarians; Semi: semi-vegetarians.

Compared with the NV group, 33, 5, and 13 significant DE miRNAs were identified in lacto, semi, and vegan groups, respectively (Supplementary Table 4). The volcano plots showing DE miRNAs for each comparison are presented in Figure 3A–C, with significant DE miRNA (i.e., the top 10 DE miRNA by |log2FC| if more than 10 significant DE miRNAs were identified) highlighted. Compared to vegetarians as a whole, we observed distinguishable DE miRNA distribution patterns in each vegetarian subgroup. Five of the significant DE miRNAs including miR-204-3p (padj = 0.0002, log2FC = 3.45), miR-320c (padj = 7.98E-06, log2FC = 2.24), miR-320b (padj = 0.0001, log2FC = 1.67), miR-29a-3p (padj = 0.03, log2FC = 0.84), and miR-132-5p (padj = 0.14, log2FC = −1.6) in the lacto group were also observed in vegetarians as a whole with the same directions of regulation. However, the semi group did not share any significant DE miRNAs with vegetarians as a whole, with miR-10397-5p (padj = 0.002, log2FC = 20.9), miR-6834-5p (padj = 0.17, log2FC = −15.58), and miR-449b-5p (padj = 0.18, log2FC = 15.28) top DE miRNAs. Similar to vegetarians, vegans also had significantly upregulated miR-29a-3p (padj = 0.18, log2FC = 1.13), with other DE miRNAs including miR-6834-5p (padj = 3.25E-08, log2FC = −32.99), miR-6742-5p (padj = 0.007, log2FC = −21.39), and miR-490-3p (padj = 0.003, log2FC = −18.19) being the most significant DE miRNAs. UpSet plots displaying the overlap of significant DE miRNAs between each vegetarian subgroup are shown in Figure 3G for upregulated miRNAs and Figure 3H for downregulated miRNAs. Vegetarian subgroups did not share any DE miRNAs, with 24 upregulated and 3 downregulated miRNAs exclusive to the lacto group, 2 upregulated and 2 downregulated miRNAs exclusive to the semi group, and 5 upregulated and 5 downregulated miRNAs exclusive to the vegan group. The IPA miRNA target analysis identified 3480 mRNA targets for the DE miRNAs between the lacto and NV groups (Supplementary Table 5A), 820 mRNA targets for the DE miRNAs between the semi and NV groups (Supplementary Table 5B), and 2300 mRNA targets for the DE miRNAs between the vegan and NV groups (Supplementary Table 5C). The overlapping target mRNAs between the vegetarian subgroups are shown in Supplementary Figure 2A for upregulated mRNAs and 2B for downregulated mRNAs. Although there were no DE miRNAs observed in all three vegetarian subgroups as well as vegetarians as a whole, there were 183 upregulated and 1 downregulated target mRNAs (highlighted in yellow) shared among the three subgroups. There were also 4 upregulated and 321 downregulated target mRNAs (highlighted in green) observed in all three subgroups as well as in vegetarians as a whole.

The top 10 most enriched canonical pathways across the three vegetarian subgroups’ target mRNAs are presented in Figure 3D–F, with the hepatic fibrosis/hepatic stellate cell activation, hepatic fibrosis signaling and IL-6 signaling, and osteoarthritis pathway being the most enriched in the lacto group; ephrin receptor signaling, hepatic fibrosis signaling, cardiac hypertrophy signaling, and breast cancer regulation being the most enriched in the semi group; hepatic fibrosis/hepatic stellate cell activation, GP6 signaling, hepatic fibrosis signaling, and cardiac hypertrophy signaling being the most enriched in the vegan group. The comparison analysis of the enriched pathways in lacto vs. NV, semi vs. NV, and vegan vs. NV revealed that the different regulatory effects of these three different vegetarian subgroups were most significant on pathways such as G Beta Gamma signaling pathway, cardiac hypertrophy signaling, and hepatic fibrosis signaling, among others (Fig. 3I).

### Sex-dependent circulating miRNA expression in vegetarians compared to non-vegetarians

**Figure 4.**
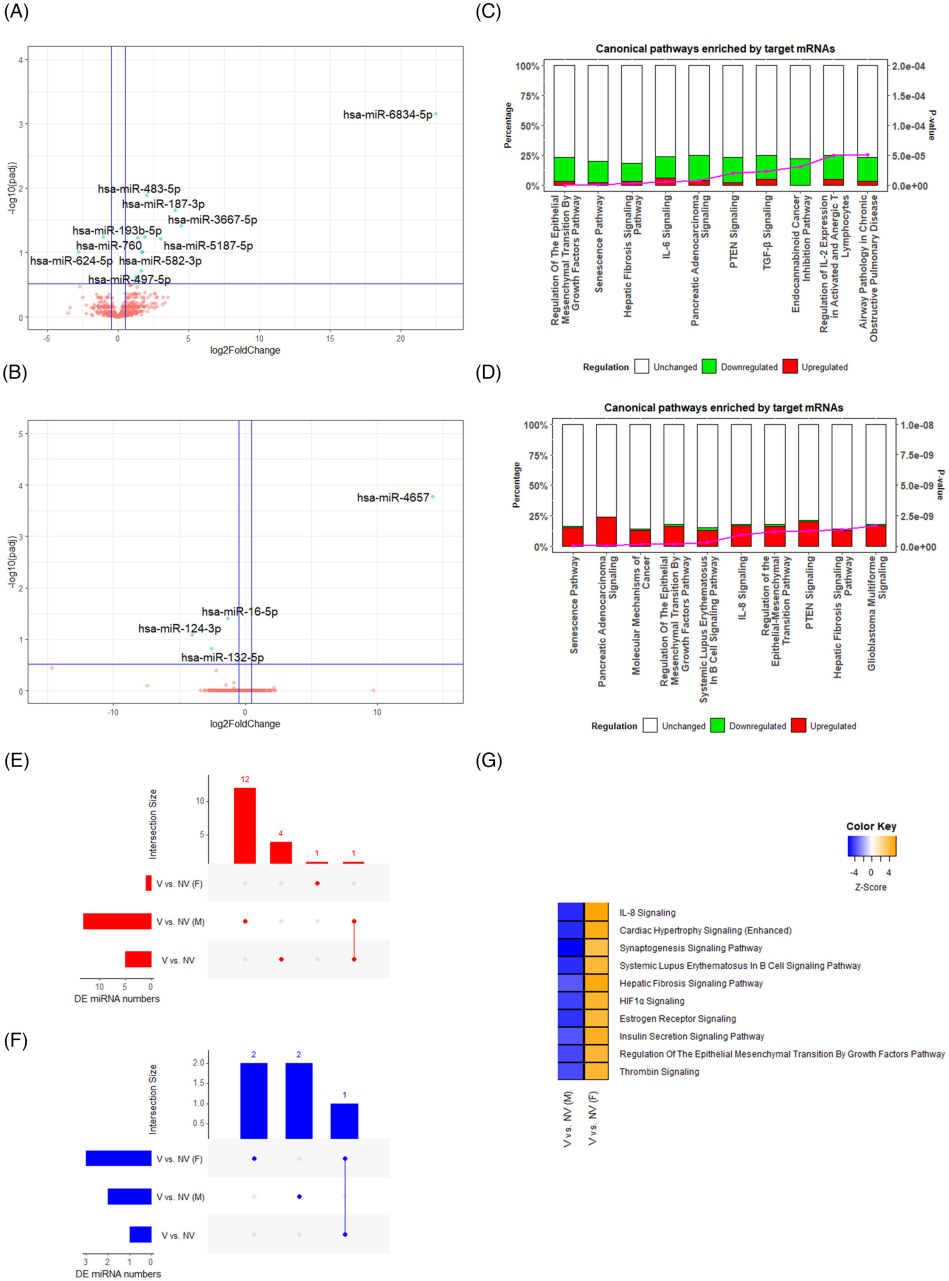
Differentially expressed miRNAs in males and females. Volcano plot showing differentially expressed miRNAs between V and NV in (**A**) males and (**B**) females. –log10(padj) ≥ 0.52 (padj ≤ 0.3) and |log2FC| ≥ 0.5 were marked as the significant threshold. The significant DE miRNAs (top 10 DE miRNA by |log2FC| if more than 10 significant DE miRNAs present) are highlighted. Bar graphs showing the top 10 enriched canonical pathways between V and NV in (**C**) males and (**D**) females. UpSet plots showing the number of significantly (**E**) upregulated, and (**F**) downregulated DE miRNAs across three comparisons (V vs. NV in males, V vs. NV in females, and V vs. NV in general), and the overlapping between each set of miRNAs. (**G**) Most distinctive enrichments in canonical pathway between male and female vegetarians. V: vegetarians, NV: non-vegetarians, M: males, F: females.

Fifteen and four significant DE miRNAs were identified between vegetarians and non-vegetarians in males and females, respectively (Supplementary Table 6). The volcano plots of DE miRNAs in males (Fig. 4A) and females (Fig. 4B) are shown with the significant DE miRNAs highlighted. As shown, miR-6834-5p (padj = 0.0007, log2FC = 22.49), miR-3667-5p (padj = 0.04, log2FC = 4.49), and miR-187-3p (padj = 0.02, log2FC = 4.06) were the most significant differentially expressed miRNA in male vegetarians compared to male non-vegetarians. miR-4657 (padj = 0.0002, log2FC = 14.24), miR-124-3p (padj = 0.08, log2FC = −4.08), miR-132-5p (padj = 0.15, log2FC = −2.54), and miR-16-5p (padj = 0.04, log2FC = −1.29) were the most significant differentially expressed miRNA in female vegetarians compared to female non-vegetarians. UpSet plots displaying the DE miRNAs of both sexes showed no common DE miRNAs, with the majority of miRNAs exclusive to either sex (Fig. 4E and F). The IPA miRNA target analysis identified 2204 mRNA targets for the DE miRNAs between V and NV in males (Supplementary Table 7A) and 1232 mRNA targets between V and NV in females (Supplementary Table 7B). UpSet plots showed that 796 commonly upregulated target mRNAs were observed in both sexes with another 2 observed in both sexes and collectively all vegetarians (Supplementary Fig. 2C). Both males and females shared 37 common downregulated target mRNAs, with another 94 observed in both sexes as well as collectively among all vegetarians (Supplementary Fig. 2D).

The top 10 most enriched canonical pathways of these two target mRNA lists are presented in Figure 4C and D. In male vegetarians, the most enriched pathways were those involved in the regulation of epithelial mesenchymal transition by growth factor, senescence, and hepatic pathways. In female vegetarians, the most enriched pathways were the senescence pathway, pancreatic adenocarcinoma signaling, and molecular mechanism of cancer. The comparison analysis of the enriched pathways between V and NV in males and in females revealed different regulatory effects on pathways, especially the IL-8 signaling, cardiac hypertrophy signaling (enhanced), and synaptogenesis signaling (Fig. 4G).

### Adherence to dietary pattern and circulating miRNA expression in vegetarians compared to non-vegetarians

**Figure 5.**
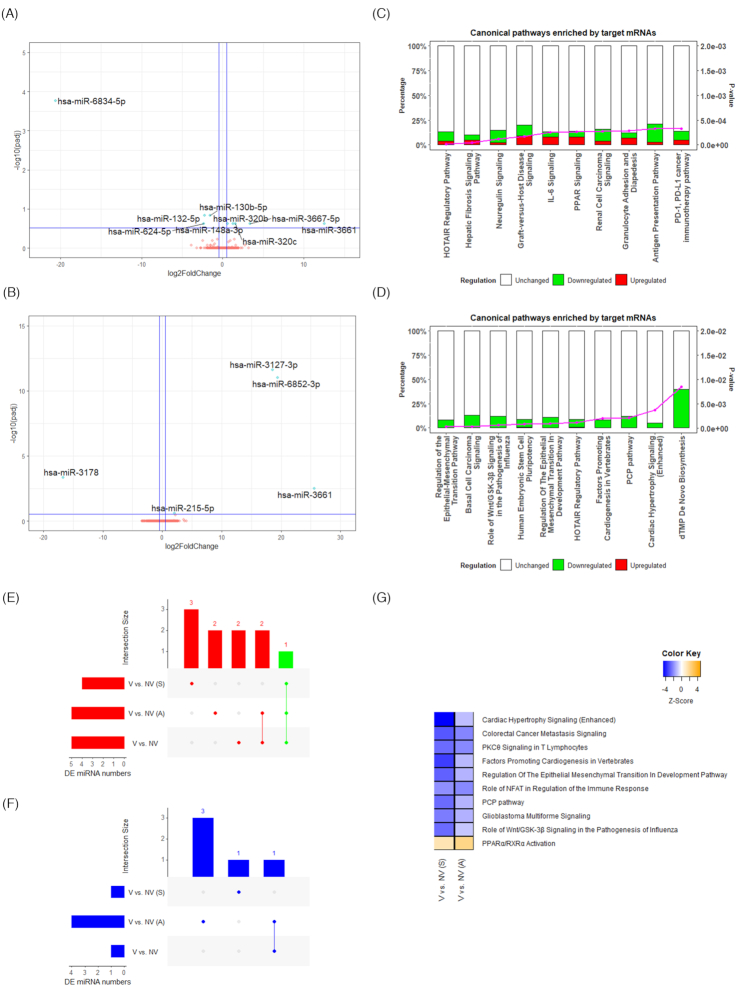
Differentially expressed miRNAs in adherers and switchers. Volcano plot showing differentially expressed miRNAs between V and NV in (**A**) adherers and (**B**) switchers. –log10(padj) ≥ 0.52 (padj ≤ 0.3) and |log2FC| ≥ 0.5 were marked as the significant threshold. The significant DE miRNAs (top 10 DE miRNA by |log2FC| if more than 10 significant DE miRNAs present) are highlighted. Bar graphs showing the top 10 enriched canonical pathways between V and NV in (**C**) adherers and (**D**) switchers. UpSet plots showing the number of significantly (**E**) upregulated, and (**F**) downregulated DE miRNAs across three comparisons (V vs. NV in adherers, V vs. NV in switchers, and V vs. NV in general), and overlapping between each set of miRNAs. The DE miRNAs shared across all three comparisons are highlighted in green. (**G**) Most distinctive enrichments in canonical pathway between adherer and switcher vegetarians. V: vegetarians, NV: non-vegetarians, A: adherers, S: switchers.

Nine and five DE miRNAs were identified between V and NV in either adherers or switchers, respectively (Supplementary Table 8). Volcano plots displaying DE miRNAs in the two groups are shown in Figure 5A and B, with the top 10 DE miRNAs highlighted. In adherers, hsa-miR-6834-5p (padj = 0.0002, log2FC = −20.6), hsa-miR-3661 (padj = 0.24, log2FC = 12.55), and hsa-miR-3667-5p (padj = 0.24, log2FC = 3.34) were among the top significant DE miRNAs. In switchers, hsa-miR-3661 (padj = 0.003, log2FC = 25.57), hsa-miR-6852-3p (padj = 9.37E-12, log2FC = 19.39), hsa-miR-3127-3p (padj = 2.47E-12, log2FC = 18.58), hsa-miR-3178 (padj = 0.0004, log2FC = −16.71), and hsa-miR-215-5p (padj = 0.24, log2FC = 2.05) were significant differentially expressed miRNAs. UpSet plots (Fig. 5e and f) showed only one upregulated DE miRNA, which was shared by all adherers, switchers, and collectively all vegetarians, while the majority of other DE miRNAs were not common to the groups. The IPA miRNA target analysis identified 1027 mRNA targets for DE miRNAs between V and NV in the adherers (Supplementary Table 9A), and 745 mRNA targets between V and NV in the switchers (Supplementary Table 9B). UpSet plots (Supplementary Fig. 2E) showed that two common upregulated target mRNAs were observed in both adherers and switchers, with another 14 observed in all adherers, switchers, and vegetarians collectively. Additionally, UpSet plots (Supplementary Fig. 2f) showed that 15 common downregulated target mRNAs were observed in both adherers and switchers, with another 1335 downregulated mRNAs observed in all adherers, switchers, and vegetarians collectively.

The top 10 most enriched canonical pathways of these two target mRNA lists are presented in Figure 5C and D. In adherers, the most enriched pathways were the HOTAIR regulatory pathway, hepatic fibrosis signaling, and neuregulin signaling. In switchers, the most enriched pathways included those involved in regulation of epithelial mesenchymal transition pathway, basal cell carcinoma signaling, as well as Wnt/GSK-3β signaling in the pathogenesis of influenza. A comparison analysis on the enriched pathways between adherers and switchers revealed that the duration of adherence to dietary patterns had similar regulatory effects on pathways such as cardiac hypertrophy signaling (enhanced), colorectal cancer metastasis signaling, and PKCθ signaling in T lymphocytes (Fig. 5G).

## Discussion

To study the impact of diet on aging and its underlying mechanisms, a quantitative and integrative biomarker is needed.^23^ A serum miRNA expression profile comparison by PCR array on a long-lived subgroup (76–92 years old) vs. shorter-lived subgroup (58–75 years old) obtained from 16 participants of the Baltimore Longitudinal Study of Aging (BLSA) identified six significantly differentially expressed miRNAs.^29^ Huan *et al*. constructed a linear miRNA age prediction model based on age-associated whole blood miRNA expression profile changes, which further highlights the potential of miRNAs as biomarkers for aging and age-associated diseases.^30^ In addition, diet-induced miRNA expression changes in humans have been investigated in several interventional studies.^31,32^ Findings from these studies support the need for the development of diet-induced circulating miRNA biomarkers for aging and age-associated diseases.

Using high throughput next generation sequencing, we identified multiple circulating miRNAs with different expression in vegetarians compared to non-vegetarians. Among the DE miRNAs in vegetarians, we observed several miRNAs that have been associated with aging or age-associated diseases in previous studies, including thrombotic diseases, neurodegenerative diseases, and age-related connective tissue disorders. Interestingly, miR-3661, the most upregulated miRNA in vegetarians, is associated with the regulation and homeostasis of protein serine/threonine phosphatase 2A (PP2A) in humans by targeting the PPP2CA gene.^33^ PP2A negatively regulates the AKT pathway, a pathway activated during aging and involved in Alzheimer's disease pathogenesis.^34–36^ Circulating miR-320b level has been reported to be upregulated in long-lived individuals in a study that compared miRNA expression of 15 centenarians and nonagenarians (mean age 96.4 years) with that of 55 younger individuals (mean age 45.9 years).^37^ We found another upregulated miRNA in vegetarians, miR-29a, which is a member of miR-29 family targeting collagens and DNA methyl transferases (DNMTs), and has been associated with aging in multiple studies. miR-29a was found to be downregulated in serum from patients with sporadic Alzheimer's disease, and its loss might contribute to increased amyloid β levels.^38^ It has also been shown that aging-induced miR-29a upregulation protected neurons from oxidative stress by regulating intracellular iron homeostasis in short-lived turquoise killifish brains. Upregulation of the miR-29 family has also been shown to preserve cardiac health during aging by limiting fibrosis and DNA methylation in the short-lived turquoise killifish.^39^ miR-320c, which was upregulated among vegetarians in our study, has been reported not only to correlate with aging of cartilage tissue,^40^ but also to inhibit the development of osteoarthritis by targeting the canonical Wnt pathway.^41^ miR-132, the only miRNA downregulated among vegetarians in our study, has been associated with multiple neurodegenerative diseases such as Alzheimer's disease and Parkinson's disease.^42^ miR-204, a miRNA inhibiting autophagy of cells, was found to be upregulated in vegetarians.^43^ The regulatory roles of these DE miRNAs as further explored by IPA suggests that they are involved in connective tissue development/disorders, such as hepatic fibrosis and osteoarthritis, both of which are classic aging-related conditions.^44,45^ Interestingly, we noted a significant downregulation in GP6 signaling, a biomarker for platelet activation, in vegetarians. Multiple studies have reported age-related increases in platelet reactivity, affecting both bleeding and thrombosis.^46^ This observation suggests a potential protective role of vegetarian diets against thrombotic diseases, which are more prevalent among older populations. Additionally, we noted the downregulation of genes involved in clathrin-mediated endocytosis in vegetarians, a pathway that could increase the likelihood of developing Alzheimer's disease by increasing amyloid-β production in neurons.^47^ In summary, our results suggest that there are various differentially-expressed miRNAs in vegetarians compared with non-vegetarians. These are mostly involved in connective tissue function, platelet activity, and neuronal function. These age-related biological processes suggest that diet may affect aging through circulating miRNA expression, and that a vegetarian diet might benefit aging and the healthspan through ameliorating connective tissue disorders, thrombotic diseases, as well as neurodegenerative diseases. However, further validation at protein and functional levels is needed to better understand or confirm the relationships between dietary patterns and aging.

Because of the non-homogenous dietary composition of vegetarians, we further categorized vegetarians into vegans, lacto-ovo-vegetarians, and semi-vegetarians to examine more closely the diet-related DE miRNA expression. We observed clear differences in DE miRNA distributions when comparing each vegetarian subgroup to non-vegetarians, indicating an averaging effect of aggregating vegetarian subgroups. The enriched cannonical pathways in these three subgroups also varied, while the most enriched pathways had certain similarities to those of vegetarians as a whole: the hepatic fibrosis pathway was the top enriched pathway in all three sub-vegetarian groups, albeit at different activation directions. Osteoarthritis and GP6 signaling were enriched in both lacto and vegan groups. It is worthy of note that IL-6 signaling, a proinflammatory pathway associated with diets high in fat and processed meat,^48^ was one of the most downregulated pathways in the lacto group. The regulations on G Beta Gamma signaling, cardiac hypertrophy signaling, and hepatic fibrosis were the most distinct among three vegetarian subgroups. Interestingly, we noticed that semi and vegan subgroups shared similar regulation patterns of these pathways, while the lacto subgroup showed opposite effects. While it might be possible that this trend was caused by different sample sizes of each dietary subgroup, the impact of the difference between vegetarian dietary pattern subgroups is still worth exploring. Among the most distinctively enriched pathways, G protein-coupled receptor (GPCRs) signaling is involved in a variety of cellular processes such as K^+^ and Ca^+^ channel regulation, cell proliferation, and survival. Studies have shown age-dependent changes in GPCR activity and their involvement in aging-associated diseases such as cancer, neurodegenerative diseases, and cardiovascular diseases.^49^ The opposite regulation in this pathway could be a result of a higher calcium intake and absorption among lacto-ovo-vegetarians.^50,51^ We also observed that the regulation direction of synaptogenesis signaling, a pathway associated with age and cognitive deficits such as Alzheimer's disease and Parkinson's disease,^52,53^ differed between lacto-, semi-vegetarians, and vegans. Synaptogenesis is affected by brain levels of key nutrients, and is dependent on endogenous synthesis and dietary intake.^54^ Studies have shown that diets high in animal fat and cholesterol increase the risk of neurodegenerative diseases, while plant-based diets rich in antioxidants lower the risk.^55,56^ Deficiencies in vitamin B12 and omega-3 fatty acids, mainly sourced from meat or fish, also increase the risk of neurodegenerative diseases. One possible explanation for the observation of upregulated synaptogenesis among semi-vegetarians and vegans is that animal products make up only a small proportion of their diet compared to lacto-ovo-vegetarians, while vitamin B12 and omega-3 fatty acid could be obtained from dietary supplements.^50,51^ Furthermore, lacto-ovo-vegetarians might have higher animal fat and cholesterol intake from eggs and milk.^50,51^ In summary, our results suggest that differences in intake of specific nutrients among lacto-, semi-vegetarians, and vegans lead to distinctive modulation of miRNA expression related to aging and the healthspan. This finding is consistent with observations from previous AHS-2 studies, which found that correlations between various health outcomes and mortality varied in vegans, lacto-ovo-vegetarians, and semi-vegetarians.^16,18^ Our study suggests that these three vegetarian subgroups had distinctive circulating miRNA profiles, including aging-related miRNAs, which may shed some light on the potential mechanisms linking vegetarian dietary patterns to improved health outcomes. Further research is needed on mechanisms by which specific dietary factors contribute to miRNA regulation and the subsequent modulation of corresponding cellular functions.

Other than the previously reported sexual dimorphism in miRNA regulation, different lifestyles and stress levels associated with each sex could be responsible for the distinctive DE miRNA expression observed in this study. Differential analyses performed within each sex showed different diet-modulated miRNA expression in males and females. The comparison of enriched canonical pathways between the two sexes suggests opposite regulation of pathways involved in aging processes. First, we observed that the senescence pathway, a hallmark of aging,^57^ was one of the most inhibited pathways in male vegetarians compared to male non-vegetarians, while in females, the senescence pathway was activated in vegetarians. Moreover, the regulation of epithelial mesenchymal transition by growth factor (a contributor to tissue fibrosis during aging^58^), cardiac hypertrophy signaling (a hallmark of cardiac aging^59^), and hepatic fibrosis signaling (a key component in liver aging^45^) were all inhibited in male vegetarians compared to male non-vegetarians. However, in females, these three pathways were activated in vegetarians. We also identified inflammatory pathways that were regulated differently between the two sexes; IL-6, IL-8, and TGF-β signaling pathways were downregulated in male vegetarians, while IL-8 signaling was upregulated in female vegetarians. As reported in previous studies, vegetarianism is associated with lower inflammatory biomarker expression in the serum and reduced gut inflammation.^60,61^ Therefore, it would be worth exploring whether the vegetarianism-induced anti-inflammatory effect is sex-dependent. We also observed similar opposite regulation of pathways involved in neuronal function and cancer, both closely involved with aging, in males and females, which further support the sex-dependent regulation of diet on circulating miRNA expression. Our results are consistent with previous AHS-2 findings that associations between vegetarian diets and mortality appeared to be stronger in males.^18^ While further validation is required, the sex-specific influence of diet on circulating miRNA regulation should be considered in the development of candidate biomarkers for aging and the healthspan.

By comparing current dietary habits with baseline dietary patterns, we were able to categorize participants based on their adherence to dietary patterns. We identified distinctive circulating miRNA expression in vegetarians compared to non-vegetarians by adherers or switchers. These two groups shared a large portion of mRNA targets, which were consistent with vegetarians collectively. Although the two groups had differences between the 10 most highly enriched canonical pathways, our comparison analysis suggested the same direction of regulation in the majority of the pathways. For example, multiple immune response-related pathways were inhibited in vegetarians of both groups, for example PKCθ and NFAT signaling, albeit at different intensities. Similar inhibition patterns were observed in cardiovascular signaling-related pathways and cancer-related pathways in both groups. It is worth pointing out that PPARα/RXRα activation, a transcription factor with anti-inflammatory and anti-oxidative effects, was upregulated in both groups. Despite the similarities between adherers and switchers, switchers had less significant DE miRNAs and enriched pathways, possibly because of the shorter time following a vegetarian diet. Another factor considered when looking at the distinction between these two groups was the significant difference (*P* = 0.03) in the adherer ratios between vegetarians and non-vegetarians. Based on this comparison, the effect of diet-induced circulating miRNA regulation seemed to be established and stable within a relatively short duration (less than 10 years), although further validation is required. Our study showed similarities and differences in diet-modulated circulating miRNA expression resulting from the stability of dietary patterns, which may serve as a guideline for developing potential miRNA-based aging and healthspan biomarkers.

Exploiting the ASH-2 cohort, our study is one of the first to investigate the effect of vegetarian diets on circulating miRNA expression among a larger number of subjects than previous studies.^62–64^ By analyzing miRNA expression in lacto-, semi-, and vegan groups separately, we detected distinct plasma miRNA expression. Our study also provided potential mechanisms for the longer life expectancy associated with residence in a Blue Zone, which has important implications for healthspan throughout the United States and elsewhere in the world. We recognize the possible variability that might have been created in our study because of the time between subjects’ recruitment and blood sample processing for our companion studies. Thus, it should be noted that biological variability and technical variation (e.g. miRNA-seq library constructions) might have impacted the number of significant miRNAs identified. Furthermore, it is anticipated that a larger sample size could allow identification of more significant circulation miRNAs modulated by vegetarian diets. Our findings from this study are subject to further validation in another cohort.

## Conclusions

Using deep sequencing, this study revealed the modulating effects of long-term vegetarian diets on circulating miRNA expression in the AHS-2 cohort. We identified several dietary-regulated circulating miRNAs associated with aging and age-associated diseases. In particular, several miRNAs were associated with neurodegenerative diseases and connective tissue diseases, suggesting a potential protective role of vegetarian diets against these age-related disorders. These significant miRNAs could serve as potential biomarkers for studying vegetarian diet-modulated longevity in future dietary intervention studies. Our results demonstrated influences of specific dietary patterns, sex, as well as stability of dietary pattern on diet-modulated miRNA expression.

## Materials and methods

### Study subjects

The Adventist Health Study 2 (AHS-2) is a population-based longitudinal study of more than 96  000 male and female members of the Seventh-day Adventist church in the USA and Canada, recruited between 2002 and 2007.^17,18^ The cohort is healthy: at baseline, high proportions reported being in excellent health; 45% of cohort members follow vegetarian diets,^65^ the non-vegetarians consume relatively lower amounts of meat compared to the general population,^51,66^ only 1.1% are current smokers and 6.6% currently drink alcohol.^17^ In 2016, the AHS-2 Cognitive and Neuroimaging (AHS-2-CAN) sub-study^67^ identified 2685 members of the cohort for whom study records indicated were 60 years or older, community-dwelling, and living within 75 miles of Loma Linda University (LLU). During 2016–2018, 199 were reached by telephone and invited to participate in the study. Of those, 168 (84%) agreed to participate and were screened for eligibility. Two did not meet inclusion criteria for being proficient in writing, speaking, and understanding English. Twelve changed their mind, one could not be scheduled, 12 could not be reached again, and five postponed participation for travel or health-related reasons. One hundred and thirty-six otherwise healthy adults were enrolled in the study and attended an in-person visit at our study clinic where a blood sample was obtained. Participants with health reasons to contraindicate blood withdrawal (e.g. severe anemia) or medical conditions that could adversely impact cognitive function were excluded. The first 96 participants for whom a blood specimen was obtained were included in the current analysis.

### Assessment of diet and baseline differences between dietary groups

Dietary intake was initially assessed at enrollment (baseline) in the AHS-2 cohort (i.e. during 2002–2007) using a self-administered food frequency questionnaire (FFQ), which assessed over 200 food items. Participants reported their average frequency of intake and usual serving size of food items consumed during the past year using predefined frequency categories according to the food under evaluation. The reported frequency of specific animal-based foods was used to define dietary patterns, including meats (meat + poultry), fish, and dairy (dairy products + eggs). Vegetarians at baseline were defined as (a) consuming meats, fish, and dairy < 1 time/month (vegan), (b) consuming dairy ≥ 1 time/month and meats, or fish < 1 time/month (lacto-ovo vegetarian, “lacto”), (c) consuming fish ≥ 1 time/month, no limits on dairy, and meats < 1 time/month (pesco-vegetarian, “pesco”), or (d) consuming meats ≥ 1 time per month with the sum of meats and fish < 1 time per week (semi-vegetarian, “semi”). Otherwise participants were classified as non-vegetarian if they consumed meats ≥ 1 time per week. Based on year of enrollment in the cohort, the average total number of years in reported dietary patterns was 21 years for vegans, 39 years for lacto-ovo-vegetarians, 19 years for pesco-vegetarians, 24 years for semi-vegetarians, and 48 years for non-vegetarians. Dietary habit was assessed at the time of blood sample collection (i.e., 2016–2018) using a brief questionnaire that asked participants to recall the frequency of consumption of five animal-based foods (meat, poultry, fish, eggs, and dairy). Current dietary habits were defined as above for vegetarian and non-vegetarian groups. In analyses, semi- and pesco-vegetarian were grouped together as semi-vegetarian because of the small number of subjects in each individual dietary pattern. Dietary patterns at baseline were compared with dietary habits reported at the time of blood collection, and participants were categorized based on the concordance between the two as follows: participants who maintained their baseline dietary pattern were assigned to the "adherer" group; participants who switched dietary patterns between baseline and current were assigned to the "switcher" group. The mean duration of adherence to the dietary pattern for the adherer group was > 19 years, while for the switcher group this was < 10 years. Analysis of variance (ANOVA) was used to examine differences in continuous variables for age at baseline and body-mass index (BMI) and chi-square tests for categorical variables for sex and long-term dietary pattern at baseline between participants following lacto-ovo-vegetarian, semi-vegetarian, vegan, or non-vegetarian dietary patterns. Similarly, ANOVAs and chi-square tests were used to examine differences in these variables comparing participants following any vegetarian compared to non-vegetarian dietary patterns. A statistical significance level was set to *P* ≤ 0.05. All tests used a null hypothesis of no difference in stated parameters between dietary groups. All procedures involving research study participants were approved by the Institutional Review Board at Loma Linda University. Written informed consent was obtained from all participants.

### Plasma isolation

A sample (8 ml) of peripheral blood was drawn into a BD Vacutainer Mononuclear Cell Preparation Tube (CPT) with sodium citrate by a trained phlebotomist. The CPTs were processed within 2 hours of the blood draws following the manufacturer's protocol. For each sample, 2 ml of plasma was collected from the upper clear layer after centrifugation of CPTs at 1300 g for 30 minutes at room temperature. The isolated plasma was spun at 12 000 g, 4 °C for 15 minutes to remove cells and debris. Specimens were then aliquoted into 2 ml Eppendorf tubes, 500 μl in each tube, and frozen at -80 °C.

### Circulating miRNA extraction and quantification

Circulating miRNAs were extracted from 200 μl plasma using Qiagen serum/plasma miRNA kits (Qiagen, Hilden, Germany) following the manufacturer's protocol. miRNAs were eluted in 14 μl RNase-free water and quantified using Qubit 3.0 by miRNA assay. miRNAs were then stored at -80 °C until library construction.

### miRNA sequencing

miRNA-seq libraries were constructed using either the NEXTflex Small RNA-Seq Kit v3 (Bio Scientific, Austin, TX) for sample IDs 003 to 032, or QIAseq miRNA library kit (Qiagen) for sample IDs 033 to 107, following the manufacturers’ protocols, respectively. Briefly, 3’ and 5’ adapters were added to small RNAs present in the plasma sample in this order. Reverse transcription was performed to convert the target small miRNAs into cDNAs. An assigned index was given to each sample for multiplexing. A 22-cycle of PCR amplification was performed. After size selection by magnetic beads, DNA fragments with the correct insert sizes were selected for the miRNA-seq library. Libraries were quantified using Qubit 3.0 HS dsDNA assay (Thermal Fisher Scientific, Waltham, MA). Library size and quality were examined using the TapeStation 2200 (Agilent, Santa Clara, CA). The miRNA-seq libraries were sequenced, 76 bp, single-end, on an Illumina NextSeq 550 (Illumina, San Diego, CA) with a final loading concentration of 2.1 pM.

### miRNA mapping and counting

All sequencing data were demultiplexed using bcl2fastq (Illumina), and the fastq files were processed using the miRNA-seq pipeline of Partek Flow software, version 7.0 (Partek, St. Louis, MO) following the user's manual. Briefly, the adapters from the 3’ end were clipped using Cutadapt. Reads shorter than 15 nts were discarded, and the bases were trimmed off both ends of remaining reads with a Phred quality score cutoff of 30. The remaining reads were aligned to human genome reference 38 (hg38) using Bowtie2 aligner. A seed mismatch limit of 1 and minimum seed length of 10 were used. All uniquely aligned reads were annotated and quantified using miRBase mature microRNAs version 22. Only miRNAs with more than 10 copies were counted.

### Differential expression analysis

The differential expression analysis was performed using ‘DESeq2’ R package.^68^ Non-vegetarians were used as the control group to identify differentially expressed miRNAs (DE miRNAs) in the vegetarian groups. The DE miRNAs with a FDR adjusted p value (padj) ≤ 0.3, calculated by the Wald test, and absolute log2FC ≥ 0.5 were considered statistically significant.^68^ A differential analysis was conducted between the vegetarian (V) group (vegan, lacto-vegetarian, and semi-vegetarian combined) and the non-vegetarian (NV) group (control). Differential analyses were then conducted comparing vegan vs. NV, lacto vs. NV, and semi vs. NV. To explore the influence of sex on diet-induced miRNA regulation, the dataset was split into males and females, and a differential analysis for V vs. NV was then performed by sex. To study the influence of adherence to dietary patterns, a differential analysis was conducted for V vs. NV in adherers and switchers separately. Figure 1 summarizes the differential expression analysis.

### Target prediction and pathway analysis

Ingenuity pathway analysis (IPA) software (Ingenuity Systems, Redwood City, CA) was applied to identify target mRNAs, enriched canonical pathways, regulatory networks, and biological functions of the differentially expressed miRNAs. miRNA target prediction was performed using IPA microRNA Target Filter to generate mRNA targets for the DE miRNA lists. Both experimentally observed and predicted with high confidence targets were considered based on four algorithms (TargetScan, TarBase, miRecords, and Ingenuity Knowledge Base). The mRNA targets generated were each assigned an artificial log2FC value, equal to -1 × the miRNA log2FC value. The log2FC of target mRNA was negative if the miRNA was upregulated, and positive if the miRNA was downregulated. Of note, these assignments followed the assumption that miRNAs and their targeted mRNAs had opposite directions of expression. The target mRNA lists were then subject to the core analysis tool of the IPA. In canonical pathway enrichment diseases and functions prediction, the statistical significance of association between genes, pathways, and networks was reflected by an overlapping *P*value calculated using Fisher's exact test.^69^*P*  ≤ 0.05 was considered to be statistically significant. In activation state prediction (up/downregulated), the statistical measure of the match between the expected relationship direction and observed gene expression was reflected by a z-score, with a z-score ≥ 2 considered activated, and z-score < 2 considered inhibited.

## Supplementary Material

pbaa037_Supplemental_Files

## Data Availability

The sequencing data have been uploaded to SRA (Sequence Read Archive) under BioProject accession PRJNA639807.
