# Supplementary figures and images for: Vegetarian diets, circulating miRNA expression and healthspan in subjects living in the Blue Zone
Source: Precis Clin Med. 2020 Oct 23;3(4):245–59. doi: 10.1093/pcmedi/pbaa037 (PMC7757436; doi:10.1093/pcmedi/pbaa037)

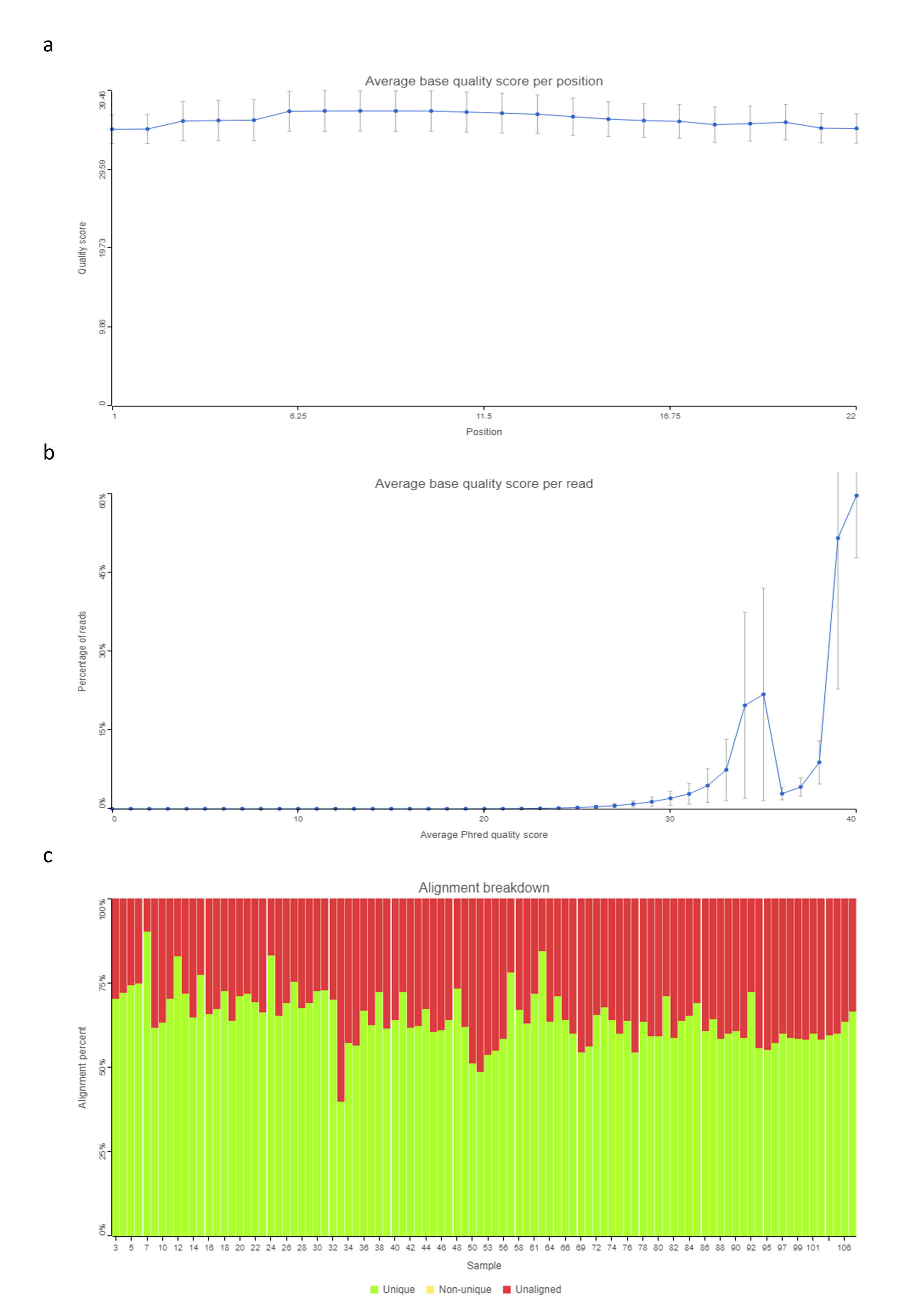

Supplement: pbaa037_Supplemental_Files [file pbaa037_supplemental_files.zip › Supplementary Fig. 1.png]

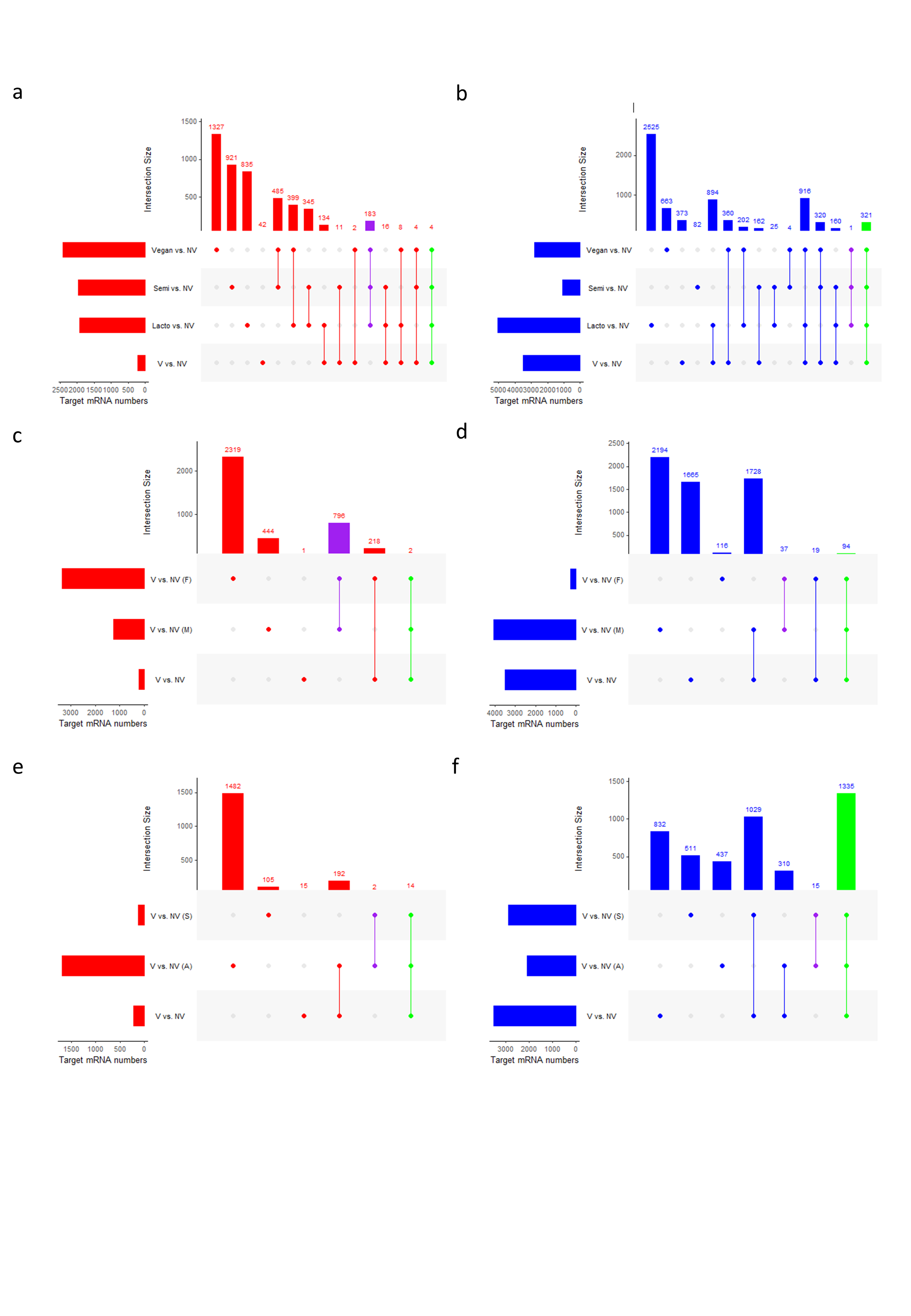

Supplement: pbaa037_Supplemental_Files [file pbaa037_supplemental_files.zip › Supplementary Fig. 2.png]
